# Unlocking the therapeutic potential of *Saussurea costus*: purification and functional characterization of α-amylase inhibitors

**DOI:** 10.3389/fbioe.2025.1535751

**Published:** 2025-01-27

**Authors:** Imen Ben Abdelmalek, Tomather A. A. Alhmdi, Abir Ben Bacha, Najeh Krayem

**Affiliations:** ^1^ Department of Biology, College of Science, Qassim University, Buraydah, Saudi Arabia; ^2^ Biochemistry Department, Science College, King Saud University, Riyadh, Saudi Arabia; ^3^ Laboratory of Biochemistry and Enzymatic Engineering of Lipases, National Engineering School of Sfax, University of Sfax, Sfax, Tunisia

**Keywords:** *Saussurea costus*, α-amylase, enzyme inhibition, diabetes mellitus, stability, antibiotic agent, cytotoxicity

## Abstract

**Introduction:**

Regulating the catalytic activity of alpha-Amylase enzymes can decrease glucose production during the postprandial phase, potentially offering therapeutic benefits for diabetes. This research aimed to assess the inhibition of α-amylase using crude extracts from *Saussurea costus,* a medicinal plant traditionally used for treating diabetes and its associated complications.

**Methods:**

Two novel potent proteinaceous amylase inhibitors: ScAI-R and ScAI-L were purified and characterized from *Saussurea costus* roots and leaves.

**Results:**

The pure inhibitors exhibited an apparent molecular weight of about 16 kDa and a high N-terminal sequence identity (81%) with the monomeric α-amylase inhibitors from *Kengyili amelanthera* and *Triticum dicoccoides*. In addition to their significant stability at extreme pH values (2.0–12.0) and temperatures (100°C), the structural integrity of both inhibitors was remarkably enhanced in the presence of divalent cations such as Mg^2+^, Ca^2+^, and Hg^2+^ at 5 mM. Interestingly, the half-maximal inhibitory concentrations of ScAI-R (IC_50_ = 23 μg/mL) or ScAI-L (IC_50_ = 28 μg/mL) against human salivary amylase against were comparable to that of the standard drug acarbose (IC_50_ = 23 μg/mL). Both purified inhibitors acted as non-competitive inhibitors with K_i_ values of 0.38 and 0.32 µM, respectively, and displayed the highest affinities towards human salivary and pancreatic α-amylases (up to 90% inhibitory activity) and, to a lesser extent, porcine pancreatic α-amylase (∼70% inhibitory activity). Furthermore, these inhibitors exhibited efficient antimicrobial activities against Gram (−) and Gram (+) bacteria, as well as fungal strains. Cytotoxicity towards the human cancer colorectal cells LoVo and HCT-116 with an IC_50_ of up to 50 μg/mL was also observed.

**Discussion:**

Thus, *Saussurea costus* α-amylase inhibitors could be potential candidates for hyperglycemic control in diabetic and colorectal cancer patients.

## 1 Introduction

Diabetes mellitus is a chronic metabolic disorder marked by high blood sugar levels resulting from insufficient or ineffective insulin production. This condition can be managed through various treatments, including insulin therapy, medication, and dietary adjustments. Without proper management, diabetes can lead to serious complications or even premature death (Erel et al., 2011; Mohammed et al., 2022). People with diabetes can take specific steps, such as losing weight and increasing physical activity, to manage the disease and reduce their risk of developing complications (Alsaadi, 2018; Rejab and Ksibi, 2019).

The International Diabetes Federation (IDF) estimates that 17.7% of Saudi Arabia’s adult population has diabetes, ranking it as having the second-highest prevalence regionally and globally (Alsaadi, 2018). According to the IDF, diabetes was responsible for 4.2 million deaths in 2019. Additionally, 463 million adults aged 20 to 79 are currently living with the condition, incurring a global healthcare cost of USD 720 billion. Projections indicate that the number of people with diabetes could rise to 700 million by 2045 (Mohajan and Mohajan, 2023). Numerous pharmaceuticals have been developed and employed to lower the blood glucose level and manage diabetes, including sulfonylureas, biguanides, and glucagon-like peptide 1 analogs, each targeting different mechanisms that disrupt glucose metabolism (Nyakudya et al., 2020). However, the side effects of these medications are concerning, highlighting the continued need for innovative and effective antidiabetic treatments. Furthermore, novel treatment approaches involving nanotechnology-based therapies have been emerging (Najjaa et al., 2020). For many decades, herbal medicines known for their hypoglycemic properties and ability to produce antidiabetic effects have been widely used for the treatment of various metabolic diseases, such as diabetes (Rabia et al., 2012; Houicher et al., 2016). Indeed, a wide variety of medicinal plants extracts and their secondary metabolites have been reported to exhibit anti-diabetic effects, primarily through the inhibition of α-amylase (α-A) or α-glucosidase (α-Glu). Moreover, these two enzymes, catalyze the breakdown of starch and glycogen, respectively, leading to an increase in the postprandial blood glucose level (Ayad et al., 2022). Because of its significant role in glucose production in the human body, α-amylase (α-A) has been a subject of research interest for many decades. Indeed, the enzyme can serve as a target for potential inhibitor agents, reducing carbohydrate metabolism and consequently decreasing and stabilizing the postprandial blood glucose level (Rejab and Ksibi, 2019; Ayad et al., 2022). Due to their lower costs, minimal side effects and easy availability, as well as their effective reduction of postprandial hyperglycemia without causing hypoglycemia, medicinal plant extracts are gaining increasing attention as natural alternatives and complementary therapies for diabetes management. Various groups of secondary metabolites, such as proteins, flavonoids, polysaccharides, phenolic compounds have been recognized as potential inhibitors of the α-amylase enzyme (Rejab and Ksibi, 2019; Nyakudya et al., 2020; Najjaa et al., 2020). As part of natural defense mechanism against diseases and pests, plant proteinaceous α-amylase inhibitors (α-AIs) are widely present in cereals, such as wheat (*Triticum aestivum*) (Møller and Svensson, 2022) and barley (*Hordeum vulgareum*) (Ashouri et al., 2015), as well as in leguminosae, such as pigeonpea (*Cajanus cajan*) (Gadge et al., 2015) and cowpea (*Vigna unguiculata*) (da Silva et al., 2020).

Based on their tertiary structure, plant proteinaceous α-AIs are classified into six classes: knottin-like, thionin-like, CM-proteins, Kunitz-like, lectin-like, and thaumatin-like. Structurally, α-AIs can be monomeric with molecular masses of 5 (Naili et al., 2010), 9 (Al-Onazi et al., 2021a), or 13 kDa (Antonelli et al., 2020); dimeric (homo and heterodimeric) (∼26 kDa) (Antonelli et al., 2020; Angulo and Lomonte, 2009); or tetrameric (∼50 kDa) (Chanda et al., 2018). The knottin-like, Kunitz, and thaumatin-like types only inhibit insect amylases, whereas the other types inhibit both insect and mammalian amylases (Chanda et al., 2018). However, a wide variability in the inhibitory actions of these plant-derived α-AIs has been described, often accompanied by low stability. Inhibition can occur through the binding of an α-AI with the enzyme’s active site via hydrogen bonds, with assistance from water molecules, or through a combination of hydrogen bonds and networks (Ayad et al., 2022). Low-molecular-weight α-AIs obstruct the amylase enzyme’s substrate binding site by creating steric hindrance, which interferes with substrate accessibility. Conversely, high-molecular-weight inhibitors interact with specific amino acids at the enzyme’s active site, thereby preventing its activity (Li et al., 2021).

Several crude medicinal plant extracts, such as *Juglansregia* and *Urtica dioica, Fiscus racemose*, *Aloe vera* leaf gel, and *Salvia officinalis*, have been extensively investigated to determine their potential hypoglycemic activities (Sarray et al., 2013). However, the development of antidiabetic drugs necessitates purification, biochemical and physiological characterization of the bioactive molecule. Few plants have been scientifically validated in terms of their potential and safety through preclinical and clinical assays (Sarray et al., 2013). Therefore, new plant sources should be investigated to find safer solutions for diabetes treatment.

Among medicinal plants, *Sausurea costus (S. costus),* member of the Asteraceae family commonly known as *Dolomiaea costus* (Elshaer et al., 2024), are known as a popular traditional medicinal plant and source of several bioactive molecules. Their pharmacological activities are extensively documented in the literature and include anti-angiogenesis, antarthritic, anticancer, anti-inflammatory, antiviral, and hepatoprotective activities. Phytochemical compounds isolated from this plant are bioactive and are potential new drug sources (Sarray et al., 2013; Tran et al., 2019). However, few studies have been conducted on antidiabetic treatments using α-amylase inhibitors produced by *S. costus*. A preliminary study on an α-amylase inhibitor showed an inhibition percentage above 70% (ALHMDI and Ben-Abdelmalek, 2023). This study focused on the purification and biochemical characterization of two α-AIs derived from the roots (ScAI-R) and leaves (ScAI-L) of the traditional medicinal plant *S. costus*. Following their characterization, the potential antidiabetic, antitumoral, and antimicrobial properties of ScAI-R and ScAI-L were investigated. Since many bacteria and cancer cells depend heavily on glucose for energy and growth, inhibiting α-amylase can reduce glucose availability, thereby suppressing their proliferation. Specifically, human salivary and pancreatic α-amylase inhibition assays were performed to assess their effectiveness in regulating postprandial blood glucose levels, suggesting potential applications in managing hyperglycemia in diabetes and colorectal cancer. This research aimed to contribute to the scientific validation of plants in the Saudi pharmacopoeia by identifying novel bioactive compounds with therapeutic potential for emerging diseases like diabetes and colorectal cancer.

## 2 Results

### 2.1 ScAI-R and ScAI-L purification

**FIGURE 1:**
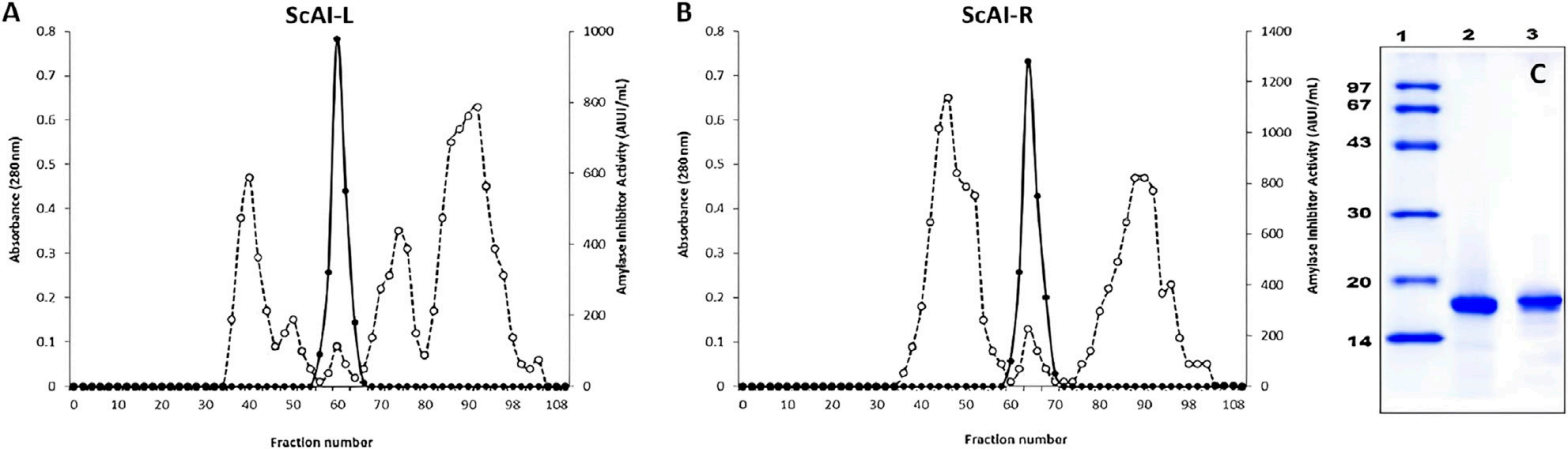
Chromatography profiles of ScAI-L **(A)** and ScAI-R **(B)** on Sepahdex-G50. **(C)** SDS-PAGE profile of purified ScAI-L and ScAI-R. Lane 1: molecular mass markers, Lane 2: ScAI-L; Lane 3: ScAI-R.

The amylase inhibitors ScAI-R and ScAI-L were purified from *S. costus* roots and leaves according to the protocol detailed in the Material and Methods section. The elution profile of the final purification step (Figure 1) shows that ScAI-R and ScAI-L were eluted at 0.54 and 0.50 column volumes (Figures 1A, B). The active fractions analyzed on SDS-PAGE indicated that both inhibitors were homogenously purified with an apparent molecular mass of about 16 kDa (Figure 1C), as previously reported for various AIs from bean (Van Meerloo et al., 2011), oats (Carmichael et al., 1988), rice (Buch et al., 2012), and sorghum (Verma et al., 2012). This suggests that ScAI-R and ScAI-L are cereal-type α-AIs, which are mostly monomeric or dimeric with molecular masses ranging from 12 to 16 kDa (Magrioti and Kokotos, 2010; Ponce et al., 2003).

Tables 1, 2 summarize the purification steps for ScAI-R and ScAI-L, respectively. The heat and acidic treatment significantly decreased the total protein content by 90% and caused an important increase in the specific activity. Purification factors of 199 and 185-fold were reached with specific inhibitory activities of 15,900 and 11,980 Amylase Inhibitor Unity/mg (AIU) for ScAI-R and ScAI-L, respectively, using human salivary amylase (Table 1).

**TABLE 1:** Purification steps for ScAI-R and ScAI-L.

Purification steps	Total activity (Units)^a^		Proteins (mg)		Specific activity (U/mg)		Activity recovery (%)		Purification Factor	
	ScAI-R	ScAI-L	ScAI-R	ScAI-L	ScAI-R	ScAI-L	ScAI-R	ScAI-L	ScAI-R	ScAI-L
Crude Extract	99,750	62,500	1,250	965	80	65	100	100	1	1
Heat and acidic treatment (100°C, pH 2.0, 15 min)	94,763	56,250	125	97.5	758	577	95	90	9.5	9
(NH_4_)_2_SO_4_ fractionation (40%–85%)	61,600		30		2,053		62		26	
Ethanol fractionation (50%–90%)		33,750		18		1,844		54		28.5
Sephadex G50	25,870	18,560	1.6	1.5	15,900	11,980	26	30	199	185

^a^ One α-amylase Inhibitory Unit (UI) is defined as the inhibitory quantity that decreases the alpha-amylase-mediated decrease in absorbance by 0.1 OD at 540 nm over 30 min.

**TABLE 2:** The effects of metal ions on ScAI-R and ScAI-L amylase inhibitor activity.

Metal ions	Amylase inhibitor activity (%)			
	ScAI-R		ScAI-L	
	1 mM	5 mM	1 mM	5 mM
MnCl_2_	102	107	99	100
CaCl_2_	120	177	110	168
FeCl_2_	90	53	82	47
NaCl	97	89	100	95
MgCl_2_	114	158	120	163
HgCl_2_	117	186	123	192
CuCl_2_	65	10	55	6
AgCl_2_	73	17	62	3

Amylase inhibitors were observed in the roots and leaves of *S. costus* in various plants, such as barley, rice and wheat, strengthening their defense against predators (Ayad et al., 2022; Naili et al., 2010; Megdiche-Ksouri et al., 2015). For instance, the α-AI isoform, one of the three bean amylase inhibitor isoforms (α-A1, α-A12, α-AIL), was found in the embryonic axes and seed cotyledons (Al-Onazi et al., 2021a).

**FIGURE 2:**
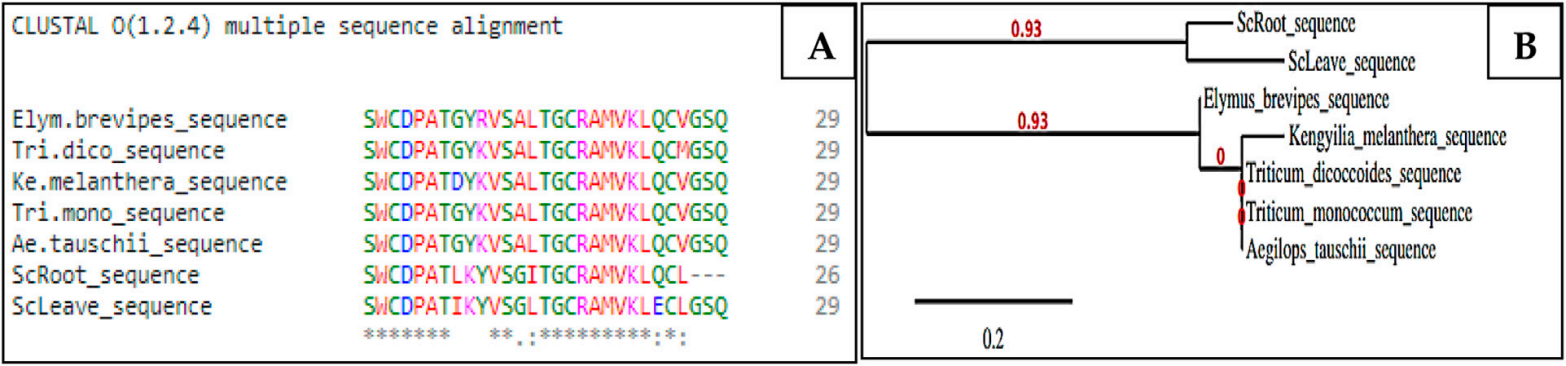
Multiple sequence alignment of the NH_2_-terminal sequences of ScAI-R and ScAI-L with the cereal-type amylase inhibitor family using the Clustal Omega tools **(A)** and phylogenetic tree **(B)**. Amino acids are color-coded to represent basic (magenta), acidic (blue), small or hydrophobic (red), and hydroxyl or amine (green). (*) Identical residues; (:) highly conserved residues; (.) significant conservation.

The NH_2_-terminal sequences of ScAI-R and ScAI-L were determined and deposed in UniProtKB under the accession numbers SPIN200023711 and SPIN200023718, respectively (Figure 2A). As illustrated in the Clustal Omega alignment, ScAI-R and ScAI-L showed high levels of sequence similarity for the determined amino acids with only three differences in residues at positions 8, 14 and 24 (Figure 2A). ScAI-R and ScAI-L exhibited around 80% identity with *Kengyiliamelanthera* (AGU17107.1) (Ali-Shtayeh et al., 1998), *Triticum dicoccoides* (ACQ83847.1), *Triticum monococcum* (ABO45935.1), *Aegilops tauschii* (ABO45963.1), and *Elymus brevipes* (AGU17133) (Abushaala et al., 2017) (Figure 2A).This high degree of sequence identity could explain the close grouping of ScAI-R and ScAI-L in the phylogenetic tree presented in Figure 2B.

### 2.2 Biochemical characterization of ScAI-R and ScAI-L

#### 2.2.1 Effect of temperature on ScAI-R and ScAI-L activity and stability

**FIGURE 3:**
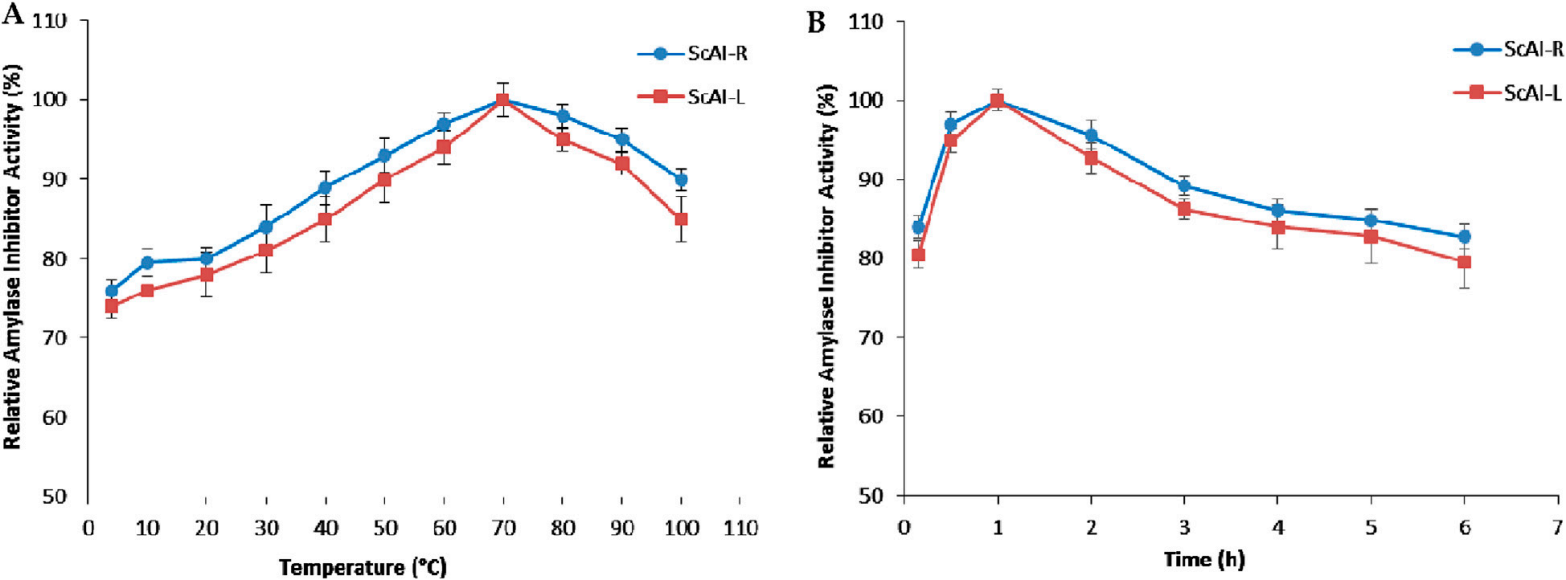
The effects of temperature on the activity **(A)** and stability **(B)** of ScAI-R and ScAI-L inhibitors. The results are expressed as the mean ± SD of three independent experiments.

Temperature and pH are interesting and promising parameters for the activity and stability of α-amylase inhibitors, allowing their use in several applications. The effects of various temperatures on purified ScAI-R and ScAI-L activity were investigated using the short standard time assay (30 min) with human salivary amylase. Interestingly, the two inhibitors were active at temperatures ranging from 4°C to 100°C with maximal activity at 70°C (Figure 3A). Interestingly, ScAI-R and ScAI-L maintained over 85% of their inhibitory activity at 100°C, indicating the maintenance of their 3D structure conformations at higher temperatures (Figure 3B). SasiKiran et al. (Eddine et al., 2017) and Hivrale et al. (Naili et al., 2010) described similar significant heat tolerance for α-amylase inhibitors from lesser yam bean (*D. esculenta*) and *Achyranthes aspera*, respectively. It is noteworthy that the remarkable thermostability of both inhibitors was also maintained even after 6 h of incubation at 100 C (Figure 3B), making them appropriate for most biotechnological applications and suggesting the potential for significant commercial exploitation of *S. costus*, as well as the potential use of ScAI-R and ScAI-L to prevent and treat obesity and diabetes. Several amylase inhibitors are thermostable, such as LDI (Limit Dextrinase Inhibitor), which displayed half-lives of 53 and 33 min at 90°C and 93 C, respectively (Sukanya et al., 2009). α-AI from wheat kernels maintained 50% of its activity after 30 min of incubation at 88.1°C (Al-Onazi et al., 2021b). Thermal denaturation of the ragi α-amylase/trypsin bifunctional inhibitor from Indian finger millet, *Eleusine coracana* (RBI), did not occur at temperatures of up to 90°C (M Hafez et al., 2022). The residual activity of the lesser yam amylase inhibitor (LYAI) decreased by up to 40% and 18% after heating for 4 h at 70°C and 80°C, respectively. At 90 C, LYAI was progressively inactivated and lost its full inhibitory activity within 4 h (Abushaala et al., 2017). Moreover, the high thermal tolerance of the barley amylase inhibitor, which lost only 50% of its initial inhibition potential after incubation at 100°C (Bouaziz et al., 2016), suggests that it can be used to support high processing or cooking temperatures and promotes its application in the development of low-glycemic-index staple foods or diabetic-friendly foods. The high thermal tolerance of highland barley-derived proteinaceous α-AIs is attributed to their 3D structures that are barely affected by temperature. It appears that the active site or the whole protein conformation of AI may be not affected by high temperatures, and the inhibitor is still able to interact with α-amylase after heat treatment (Walker and White, 2017).

**FIGURE 4:**
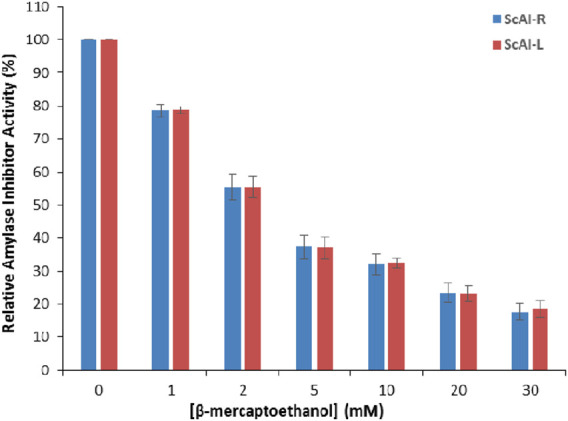
The effects of β-mercaptoethanol on ScAI-R and ScAI-L amylase inhibitor activity. The results expressed are as the mean ± SD of three independent experiments.

In order to further investigate the thermostability of ScAI-R and ScAI-L at 100°C, β-mercaptoethanol was added at concentrations ranging from 1 to 30 mM. Figure 4 shows that the relative ScAI-R and ScAI-L amylase inhibitor activities decreased by up to 50% after 30 min of incubation at 100°C in the presence of 2 mM β-mercaptoethanol using human salivary amylase. Only 20% of the inhibitory effect was maintained with 30 mM of β-mercaptoethanol, suggesting that disulfide bridges play a crucial role in the activity and stability of α-AIs (Figure 4). Similar behavior was described for the α-AIs of the cereal family, which contain five disulfide bonds and are well known to be highly thermostable (Lagrouh et al., 2017; Rane et al., 2020). In addition, RBI is a prototype of a stable monomer with 122 amino acids and five disulfide bonds that is resistant to urea, guanidine hydrochloride, and thermal denaturation (Møller and Svensson, 2022; Walker and White, 2017).

#### 2.2.2 Effects of the pH on the activity and stability of ScAI-R and ScAI-L

**FIGURE 5:**
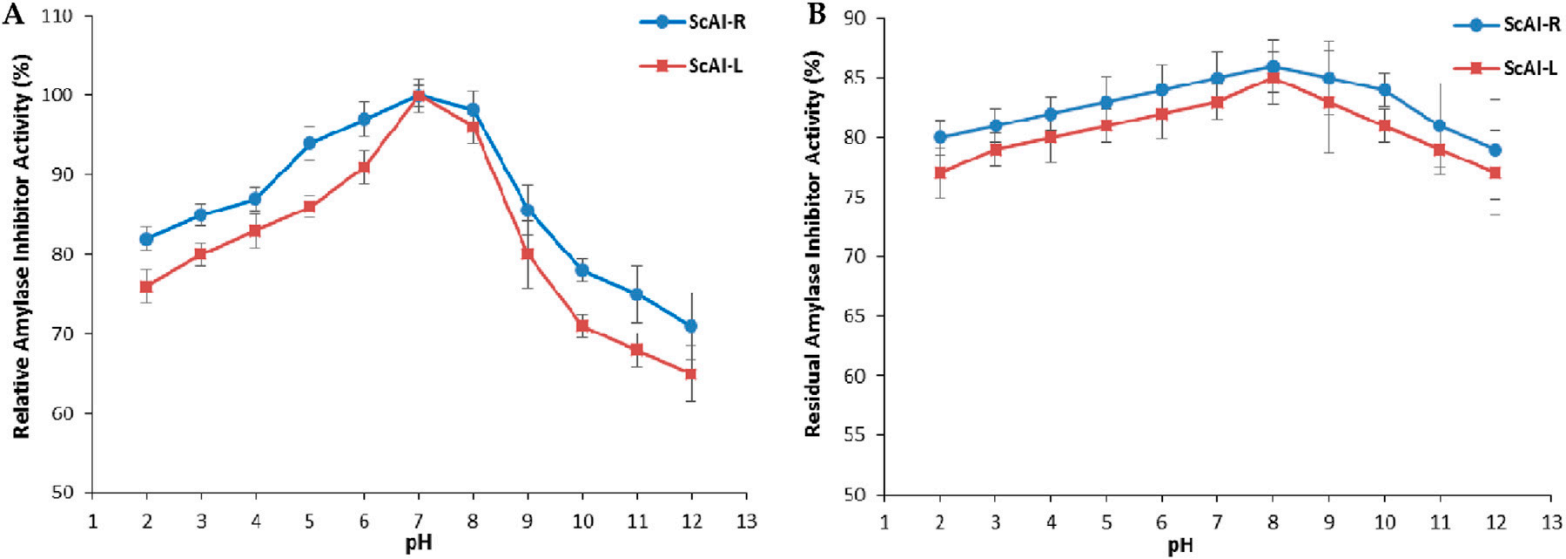
The effects of pH on the activity **(A)** and stability **(B)** of ScAI-R and ScAI-L. The results are expressed as the mean ± SD of three independent experiments.

The activity and stability of ScAI-R and ScAI-L in response to the physicochemical parameter pH were also investigated at pH values ranging from 2.0 to 12.0 using human salivary amylase. As shown in Figure 5A, a bell-shaped curve was obtained after 30 min of each α-AI pre-incubation at different pHs.

The maximum α-AI activity was observed at pH 7.0 for both inhibitors (Figure 5A). Under extreme acidic (pH 2.0) or alkaline (12.0) conditions, the α-AI inhibitory activity decreased slightly by 25% or 35%, respectively (Figure 5A). Similarly, the optimal pH for the *Moringa oleifera* leaf α-AI was 8.0 (Walker and White, 2017), while *Phaseolus vulgaris* α-AI displayed two pH optima of 5.0 and 6.9 (Hector, 1993). However, The α-AI purified from black bean exhibited its optimal activity at pH values between 4.5 and 6.0 (Ghannoum and Rice, 1999; Peddio, 2023).

Purified ScAI-R and ScAI-L were highly stable under extreme pH conditions and retained around 80% of their initial inhibitory activity after incubation at pH 2.0 or 12.0 over 24 h (Figure 5B). α-AI isolated from barley extract (Freiesleben and Jäger, 2014) or from the Beni Suef-1 and Beni Suef-5 varieties Walker and White (2017) showed a similar level of tolerance to a wide range of pH values (2.0–12.0). This unusual physicochemical stability is a remarkable feature for human salivary and pancreatic amylase inhibition, since the pH values of human gastric and salivary and intestinal fluids are 1.0–3.0 and 5.7–7.4, respectively. Therefore, *S. costus* α-AIs could resist salivary and gastrointestinal pHs and efficiently inhibit α-amylase, thereby decreasing the glycemia level. This indicates their potential use as type 2 diabetes treatments. Moreover, the stability of ScAI-R and ScAI-L across a large range of pH is an encouraging and interesting property that could be exploited in various biotechnological and pharmaceutical industries.

#### 2.2.3 Effect of metal ions on ScAI-R and ScAI-L amylase inhibitor activity

Several divalent metal ions have been described as key factors in the maintenance of the α-AIs’ 3D structural integrity. Accordingly, the effects of metal ions on the activity of the ScAI-R and ScAI-L α-AIs were determined through the addition of various ions (Ca^2+^, Mg^2+^, Na^+^, Fe^2+^, Hg^2+^, Mn^2+^, Cu^2+^, and Ag^+^) to the reaction mixture at concentrations of 1 or 5 mM using human salivary amylase. The results were compared with those of the control (100%), which was assessed under the same conditions in the absence of any metal ions. Table 2 shows that Mg^2+^, Ca^2+,^ and Hg^2+^ ions (5 mM) enhanced ScAI-R and ScAI-L activity by up to 63%, 77%, and 92%, respectively. However, the cations Fe^2+^, Cu^2+^ and Ag^+^ (5 mM) reduced the inhibitory activity by up to 47%, 6%, and 3%, respectively. At 5 mM, Mn^2+^ and Na^+^ did not affect the inhibitory activity of either studied α-AI. The best activator was Hg^2+^ (at 5 mM) for ScAI-L, which showed an increase in the inhibition potential of 92%. Similarly, at 5 mM, Ca^2+^ and Mg^2+^ improved the inhibitory activity of α-AI from *M. oleifera* L. by up to 169% and 119%, respectively, while Fe^2+^ and Hg^2+^ reduced its inhibition potential by up to 31% and 42%, respectively (Walker and White, 2017).

### 2.3 IC_50_

**FIGURE 6:**
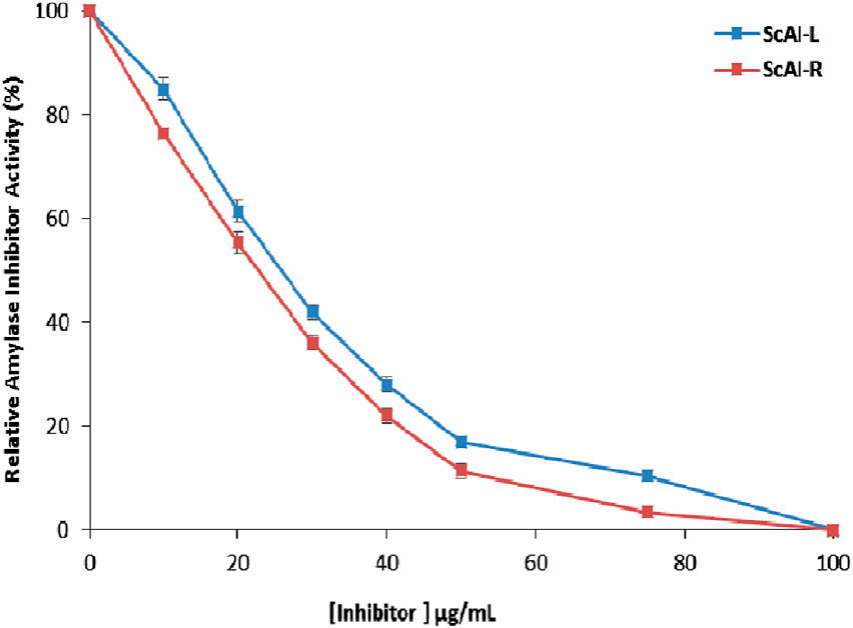
Half-maximal inhibitory concentration (IC_50_) determination for human salivary amylase inhibition by ScAI-R and ScAI-L. The results are expressed as the mean ± SD of three independent experiments.

The IC_50_ for human salivary amylase inhibition by ScAI-R and ScAI-L was determined (Figure 6). The α-amylase inhibitory activity increased with the inhibitor concentration in a dose-dependent manner (Figure 6). ScAI-R and ScAI-L strongly inhibited human salivary α-amylase activity with IC_50_ values of 23 μg/mL and 28 μg/mL, respectively. These IC_50_ values are comparable to that of the standard drug, acarbose, which has an IC_50_ of 23 µg/mL for α-amylase inhibition (Kang et al., 2010). Indeed, it has been well established that diabetes treatment with acarbose is tolerated and may improve glycemic control as a monotherapy (Kang et al., 2010). However, acarbose is associated with several negative effects such as abdominal distension, flatulence, meteorism, and mild diarrhea (Kulawik et al., 2013). Therefore, searching for new pharmacologically active, safer, specific, and effective hypoglycemic agents from natural sources that may lead to the discovery of potent and specific α-AIs continues to be an important area of investigation (Najjaa et al., 2020). ScAI-R and ScAI-L offer great potential as new antidiabetic drugs that could be effective for the management of postprandial hyperglycemia with fewer side effects compared to acarbose.

### 2.4 Kinetic parameters of ScAI-R and ScAI-L

To gain more insight into ScAI-R and ScAI-L kinetic inhibition, a Lineweaver and Burk plot of human salivary amylase was plotted using different substrate concentrations in the presence or absence of each purified inhibitor (Kang et al., 2010). Without any inhibitors, the V_max_ and K_max_ values of human salivary amylase were estimated to be 27 mmol/min/mg and 0.2 mM, respectively.

In the presence of 10 µmol of ScAI-R and ScAI-L, K_m_ did not change (0.2 mM) while V_max_ decreased to 0.055 and 0.046 mmol/min/mg, respectively. The amylase affinity was not affected, and the inhibition patterns of both purified inhibitors were of the non-competitive inhibition type with inhibition constant (Ki) values of 0.38 and 0.32 µM, respectively. Similar results were described for α-AIs from Moringa *oleifera* (Walker and White, 2017) and *P. vulgaris* (Hector, 1993) with Ki values of 21 and 23 μM, respectively. However, reversible and non-competitive inhibition was reported for the barley amylase inhibitor (Megdiche-Ksouri et al., 2015), whereas wheat kernel α-AI was shown to be a competitive inhibitor with a Ki value of 57.3 nM (Zaman et al., 2022).

### 2.5 ScAI-R and ScAI-L specificity against mammalian, bacterial and fungal α-amylases

**FIGURE 7:**
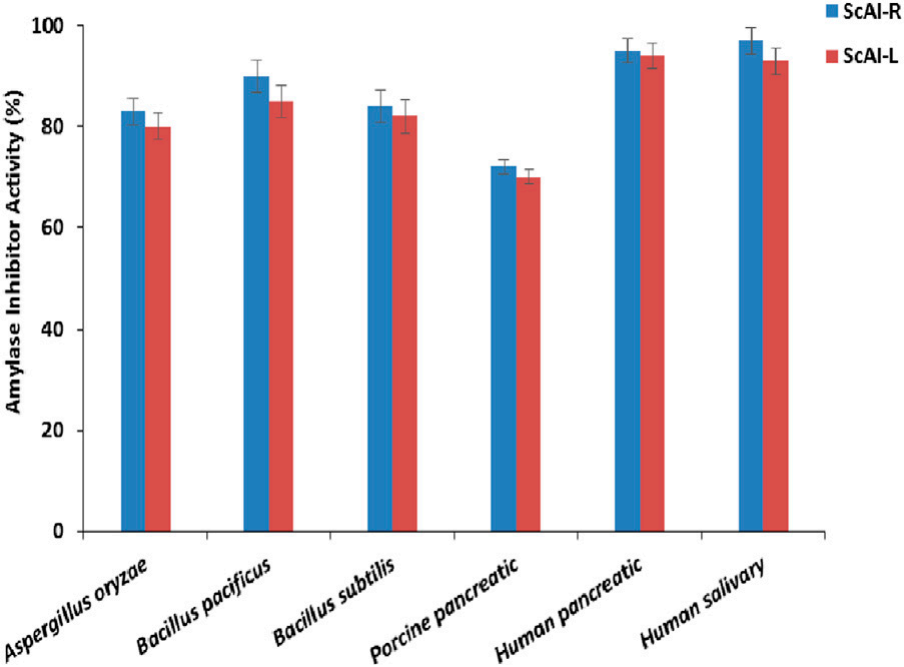
ScAI-R and ScAI-L activity against mammalian, bacterial, and fungal α-amylases. The results are expressed as the mean ± SD of three independent experiments.

Six amylases from different organisms (porcine pancreatic, human salivary and pancreatic*, Bacillus subtilis*, *Bacillus pacificus,* and *Aspergillus oryzae*) were used to determine the specificity of ScAI-R and ScAI-L (Figure 7). ScAI-R and ScAI-L exhibited the highest affinities towards human salivary and pancreatic α-amylases (90%–95% inhibitory activity), followed by α-amylase from *B. pacificus, B. subtilis* and *A. oryzae* (85, 80% and 78%, respectively) and, to a lesser extent, porcine pancreatic α-amylase (∼70%) (Figure 7). This inhibition potency variability could be explained by the difference in the amino acid compositions of human and porcine pancreatic amylases at the interface of the inhibitors, which could directly influence the specificity of the inhibition, leading to a reduced hydrogen bonding capacity in the absence of any obvious steric obstacle to the enzyme/inhibitor complex formation (EREL et al., 2012).

A high inhibition specificity against human salivary and pancreatic α-amylases has been described with α-AIs from *Moringa olifera* (Walker and White, 2017), *P. vulgaris* (KR-9) (Pisoschi and Pop, 2015), and buckwheat (Tsao, 2010). However, human saliva α-amylase activity was not inhibited by proteinaceous α-AIs extracted from chickpeas, kidney beans, maize, wheat, or millet seeds (Apak et al., 2007). *Bacillus* α-amylases were less inhibited by *Amaranthus paniculatus* α-AI, even at extremely low concentrations (Cai et al., 2003). Specificity towards *A. oryzae* α-amylase has also been shown with phenolic inhibitor compounds extracted from *A. americana* (designed by Apigenin (Hebi and Eddouks, 2016)), as well as *M. olifera* α-AI, which caused 16% inhibition of *A. oryzae* amylase (Walker and White, 2017).

### 2.6 Evaluation of the biological activities of ScAI-R and ScAI-L

#### 2.6.1 Antimicrobial activity

The search for natural therapeutic alternatives to conventional antibiotics is a growing research field, since the efficiency of antibiotics is increasingly being reduced by resistant bacteria development. The potential use of plant antimicrobial peptides/proteins, such as α-AIs, as new antimicrobial agents is promising, since they can target several cell pathogen membranes, resulting in growth inhibition or microbial death (Najjaa et al., 2020). Accordingly, the antibacterial potency of ScAI-R and ScAI-L was evaluated against Gram (+) and Gram (−) bacteria by measuring the inhibition zone diameter resulting from the inoculation of bacterial strains with purified α-AIs and by determining the IC_50_ value (Table 3). Table 3 shows that ScAI-R and ScAI-L exhibited stronger bactericidal effects against Gram (+) *E. faecalis* (ATCC 29122) and *S. aureus* (ATCC 25923) than Gram (−) *B. fragilis* (ATCC 25285) and *E. coli* (ATCC 25922). The sensitivity of the four tested strains against ScAI-R and ScAI-L was in the following order: *S. aureus* > *E. faecalis* > *B. fragilis* > *E. coli* (Table 3). *S. aureus* was the most sensitive strain towards both inhibitors with IC_50_ values of 16.5 and 12.5 μg/mL for ScAI-R and ScAI-L, respectively. The inhibitory effect against *S. aureus* strains is important, since *S. aureus* is a major human pathogen that causes a wide variety of infections and its resistance towards several antibiotics has gradually increased (Al-Onazi et al., 2021a).

**TABLE 3:** Biological activities of ScAI-R and ScAI-L: The results are expressed as the mean ± SD of three independent experiments.

	IC_50_ (µg/mL)			Inhibition zone (mm)		
	ScAI-R	ScAI-L	Ampicillin	ScAI-R	ScAI-L	Ampicillin
Bacterial strains						
*Bacteroides fragilis* (ATCC 25285)	23.5 ± 2.1	27.2 ± 1.06	16.15 ± 1.20	21 ± 1.3	23.5 ± 1.08	20.5 ± 1.4
*Enterococcus faecalis* (ATCC 29122)	19 ± 1.41	21.75 ± 1.76	12.75 ± 1.06	11 ± 1.2	12.5 ± 1.3	24.2 ± 0.7
*Staphylococcus aureus* (ATCC 25923)	16.5 ± 2.12	12.5 ± 0.70	17 ± 1.41	16.5 ± 0.9	16 ± 1.33	21.5 ± 1.4
*Esherichia coli* (ATCC 25922)	28 ± 1.41	25 ± 0.70	20.25 ± 1.76	11.5 ± 1.4	14.5 ± 1.11	22.6 ± 1.5

	IC_50_ (mg/mL)			Inhibition zone (mm)		
	ScAI-R	ScAI-L	Cycloheximide	ScAI-R	ScAI-L	Cycloheximide
Fungal Strains						
*Aspergillus niger*	6.75 ± 0.35	9.5 ± 1.41	2.65 ± 0.21	28 ± 2.1	25 ± 0.7	28 ± 0.8
*Aspergillus oryzae*	3.75 ± 0.35	4.5 ± 0.70	2.5 ± 0.28	25 ± 0.7	22 ± 1.1	30 ± 2.1
*Penicilium digitatum*	8.25 ± 0.35	6.25 ± 0.35	3.15 ± 0.21	31 ± 2.8	23 ± 1.5	22 ± 1.2

	IC_50_ (µg/mL)					
	ScAI-R			ScAI-L		
Cancer Cell Lines						
LoVo	51.5 ± 4.94			59.5 ± 3.53		
HCT-116	72 ± 4.24			58 ± 4.20		
MDA-MB	97 ± 7.07			92 ± 5.65		

Moreover, the efficient antifungal effects of the two studied inhibitors were observed with inhibition zones ranging from 22 to 31 mm in the following increasing order: *A. oryzae* (22; 25 mm) > *Aspergillus niger* (25; 28 mm) > *Penicilium digitatum* (23; 31 mm) for ScAI-R and ScAI-L, respectively (Table 3). *Aspergillus oryzae* was the most sensitive strain against both ScAI-R and ScAI-L with IC_50_ values of 3.75 and 4.5 μg/mL, respectively. This suggests the potential for a new approach using inhibitors as antifungal molecules, as previously reported for the *Fagopyrum esculentum* α-AI, which is characterized as an antifungal agent against *Aspergillus Niger* and *Aspergillus flavus* (Mourad et al., 2018). In addition to their amylase inhibitory activity, several purified peptides from plants showed antifungal properties that were explained by the permeability modification of fungal membrane spores or the plasmatic membrane (Ayad et al., 2022). Moreover, these inhibitors could impair the plasma membrane H^+^/ATPase pump, which plays an essential role in fungal cell physiology. This impairment commonly leads to cell death through the possible production of reactive oxygen species by the mitochondria and the subsequent induction of apoptosis (Najjaa et al., 2020; Ayad et al., 2022).

#### 2.6.2 Antitumoral activity

Colorectal cancer is the fourth most deadly cancer, accounting for 10.9% of all cancer cases in 2020. The emergence of drug-resistant colorectal cancer cells and the side effects of conventional treatments, especially chemotherapy, reduce patient survival and increase the probability of cancer recurrence (Faridi et al., 2017). Therefore, searching for effective and harmless new molecules for colorectal cancer treatment is a growing field. Several natural products, such as microbial or plants peptides, exhibit excellent specificity and effectiveness and minimal toxicity against tumoral cells both *in vitro* and *in vivo* (Pisoschi et al., 2009). In this context, the antimicrobial plant proteins ScAI-R and ScAI-L were evaluated to determine their antitumoral effects using the human colorectal cancer cells LoVo and HCT-116, as well as the human breast cancer cell line MDA-MB. The cytotoxic effects of the purified ScAI-R and ScAI-L at concentrations ranging from 0 to 200 μg/mL on human cancer cell lines were investigated using MTT assays. A dose–response effect was observed for the two inhibitors, with IC_50_ values of 52 ± 5 and 60 ± 3.5 μg/mL for ScAI-R and ScAI-L, respectively. However, the MDA-MB viability was slightly affected by both tested inhibitors, showing an IC_50_ value of about 90 μg/mL (Table 3). These results are in accordance with previous studies demonstrating that plant antimicrobial peptides interact with the phospholipid membranes of tumoral cells and cause cytotoxic activity (Faridi et al., 2017). Indeed, electrostatic attraction between the negatively charged phospholipids in cancer cell membranes and the positively charged plant peptides is a suggested cytotoxic mechanism. The alteration of cancer cell ion channels by these peptides was also proposed (Dai and Mumper, 2010).

## 3 Materials and methods

### 3.1 Extraction and recovery of *S. costus* α-amylase inhibitors

*S. costus* leaves and roots were purchased randomly in September 2021. The plant was identified and confirmed by Dr. Magda Gazer (Department of Biology Science, College-Qassim University). *S. costus* roots and leaves were first separated and washed in distilled water. They were then cut into small pieces to facilitate air-drying at ambient temperature before being ground. A total of 20 g of each obtained powdered tissue was homogenized in 200 mL of water, and after 24 h incubation in a rotary shaker (200 × g) at room temperature, the homogenates were filtered using a Buchner funnel and centrifuged (12,000 × g, 15 min, 4°C). The amylase inhibitor activity was then assayed in the resulting soluble crude extracts and used for further study.

### 3.2 Amylase inhibitory activity assay and specificities on amylases from different organisms

α-AI activity was investigated using starch as a substrate in accordance with Karray et *al*. (Walker and White, 2017). A starch solution was prepared in a 0.1 M Tris-HCL buffer at pH 7.0. Amylase activity was measured by incubating the enzyme solution (1 mg/mL in 0.1 M Tris-HCL buffer) with 1 mL of soluble starch solution and the same buffer at pH 7.0°C and 37°C for 5 min. Following this, 2 mL of dinitrosalicylic acid (DNS) reagent was added. The mixture was heated to boiling for 5 min, then cooled and diluted with 10 mL of water, and the absorbance was measured at 540 nm. One α-amylase unit (UI) was defined as the enzyme quantity that increased absorbance by 0.1 OD at 540 nm over 30 min. For comparative purposes, the α-AI activity was also expressed as an inhibition percentage, which was calculated by comparison with a control experiment using the following formula:

$$\text{Inhibition }\%=\left[\text{Absorbance }\left(c\right)-\text{Absorbance }\left(s\right)\right]/\text{ Absorbance }\left(c\right)\;x\;100$$

where c = control and s = sample. All experiments were carried out in triplicate.

The activities of six available amylases from different organisms (human salivary, human pancreatic, porcine pancreatic, *B. subtilis*, *Bacillus* pacificus, Aspergillus oryzae) were determined according to the protocols described by Karray et *al*. (Walker and White, 2017). Enzyme inhibition using α-AI (0.25 mg/mL) was determined after preincubation with the corresponding amylase at 1 mg/mL for 30 min. Afterwards, the remaining amylase activity was measured with the previously described protocol. The α-AI quantity that inhibits one unit of corresponding enzyme activity is referred to as an α-AI unit. The α-AI activity was also represented as an inhibition percentage, which was calculated by comparing the results to a control experiment.

The half-maximal inhibitory concentration (IC_50_) values were determined from plots of the percent inhibition versus the log inhibitor concentration and calculated via logarithmic regression analysis from the mean inhibitory values. The IC_50_ values were defined as the α-AI concentration that inhibited 50% of the human salivary amylase activity.

### 3.3 Enzymes and reagents

Human salivary α-amylase, human pancreatic amylase, porcine pancreatic α-amylase, *B. subtilis* α-amylase, *Bacillus* pacificus α-amylase, and Aspergillus oryzae were purchased from Sigma Aldrich (St. Quentin-Fallavier, France).

Chemicals were obtained from commercial sources. Chromatography material (Sephadex G-50), phosphate buffer saline (PBS, pH 7.4), sodium dodecyl sulfate (SDS), acrylamide, ammonium persulfate, N,N,N′,N′-tetramethyl ethylenediamine (TEMED), β-mercaptoethanol, and Coomassie brilliant blue R-250 were obtained from Bio-Rad.

Starch, DNS, NaCl, CaCl_2_, Tris–HCl, ethanol, bovine serum albumin (BSA), Triton X100, protein markers for molecular masses, sodium acetate, potassium acetate, and glycine were purchased from Sigma Aldrich (St. Quentin-Fallavier, France).

Dulbecco’s Modified Eagles Medium, fetal bovine serum, glutamine, streptomycin, penicillin and 3-[4,5-dimethylthiazol-2-yl]-2,5 diphenyl tetrazolium bromide (MTT) were purchased from Life Technologies (Paisley, United Kingdom).

### 3.4 Purification of *S. costus* amylase inhibitors

The purification of α-AI from *S. costus* leaves and roots was performed using a combination of heat treatment (100°C for 30 min), pH treatment (pH 2.0 for 15 min), ammonium sulfate fractionation (40%–85%), and gel filtration chromatography using the Sephadex G50 column.

According to the literature review, for some species, amylase inhibitors display high thermostability with a minimal loss of inhibitory activity (Bougandoura and Bendimerad, 2013). For this reason, the root and leaf crude extracts were first incubated at 100°C for 30 min. Then, the samples were rapidly cooled at room temperature, and thermolabile proteins were removed via centrifugation (30 min, 12,000 × g, 4°C). The pH of each resulting supernatant was decreased by up to 2.0 by the addition of HCl 1M, incubation for 30 min at room temperature, and then centrifugation at 12,000 × g for 30 min. After that, a fractionation step was performed using ammonium sulfate (40%–85%) or ethanol (50%–90%) for root and leaf crude extracts, respectively. Pellets collected via centrifugation (13,000 × g, 30 min, 4°C) were dissolved in 25 Mm Tris-HCl buffer at pH 8.0 and subsequently loaded onto a Sephadex G50 column (3 × 100 cm) pre-equilibrated with a 0.1 M Tris-HCL buffer (pH 8.0) containing 0.15 M NaCl. The same buffer was used for protein elution, and 6 mL fractions were collected at a flow rate of 0.5 mL/min. The protein elution profile was followed spectrophotometrically at 280 nm. Fractions containing amylase inhibitory activity tested using the human salivary amylase were analyzed electrophoretically with analytical polyacrylamide gel (15%) in the presence of sodium dodecyl sulphate (SDS-PAGE) (Megdiche-Ksouri et al., 2015), and pure active fractions were then gathered and stored at 4°C for further biochemical characterization and molecular analysis.

### 3.5 Protein analysis

The protein content was determined in accordance with Bradford (Bradford, 1976). The purity and molecular weight estimation of the studied amylase inhibitor were checked with 15% SDS-PAGE following Laemmli (Laemmli, 1970). The NH_2_-terminal sequence was determined by automated Edman’s degradation with the applied biosystems protein sequencer procise 492 equipped with a 140 C HPLC system (Hewick et al., 1981). The sequence homology between the α-AI amino acid sequences from *S. Costus* and those from other sources was analyzed using BLAST, UniProt (http://www. uniprot. org/), and Clustal Omega (https://www.ebi.ac.uk/Tools/msa/clustalo/). A neighbor-joining tree was also constructed with MEGA version 6 (Megdiche-Ksouri et al., 2015).

### 3.6 Effects of the pH and temperature on α-AI activity and stability

The optimum temperature for α-AI activity was determined by carrying out the enzyme assay at different temperatures (4°C–100°C) at pH 7.0. The thermal stability was determined by incubating the amylase inhibitor at 100°C for various time intervals and assayed for residual inhibitory activity under optimal pH and temperature conditions. The remaining amylase inhibitor activity was expressed as a percentage of the control using the human salivary amylase.

Aliquots (1.0 mL) of each amylase inhibitor solution in the presence and absence of 10 mM beta-mercaptoethanol (β-ME) were placed in screw-cap vials (10 × 45 mm Pyrex with Teffon-lined caps). The vials were placed in a boiling water bath at the same time. The study was carried out at pH 7.0 for 250 min with one vial of each solution being removed from the boiling water bath and subsequently cooled on ice at 30 min intervals. This step was followed by centrifugation at 12,000 × g for 15 min, and the remaining amylase inhibitor activity was expressed as a percentage of the control, as described above.

The α-AI activity was also tested in various buffers at different pHs (2.0–13.0) at 37°C. The substrate starch (1%) was prepared in the respective pH buffers as follows: 200 mM sodium acetate buffer (pH 2.0–5), 200 mM potassium phosphate buffer (pH 6.0–7.0), 200 Mm Tris-HCl buffer (pH 8.0–9.0), and 200 mM glycine-NaOH buffer (pH 10.0–13.0). The pH stability of α-AI was determined through incubation at various pH values ranging from 2.0 to 13.0 for 24 h at 4°C using the same buffers. The residual α-AI activity was determined with the standard assay method. Each measurement was performed in triplicate.

### 3.7 Metal ions influence on α-AIs’ activity

The influence of metal ions on α-AI activity was determined by adding various ions, such as Ca^2+^, Mg^2+^, Fe^2+^, Hg^2+^, Mn^2+^, Cu^2+^, Na^+^ and Ag^+^ to the reaction mixture at final concentrations of 1 and 5 mM. After incubation for 30 min at 37°C, the α-AI activity was assayed under optimal conditions.

### 3.8 Kinetic parameters of ScAI-R and ScAI-L

Human amylase activity was evaluated at various substrate concentrations with 0–1.5 mM of starch under optimal conditions (pH 7.0, 37°C) without any inhibitor or with 10 µmol of each α-AI. Measurements were recorded in triplicate, and the respective kinetic parameters, including the maximal velocity (V_max_), affinity constant (K_m_), and inhibition constant (Ki), were calculated from Lineweaver–Burk plots (Mourad et al., 2018).

### 3.9 Antimicrobial activity

The strains used were from American Type Culture Collection ATCC. The susceptibility of Gram (+) (*Enterococcus faecalis* (ATCC 29122) and *Staphylococcus aureus* (ATCC 25923)) and Gram (−) (*Bacteroides fragilis* (ATCC 25285) and *Escherichia coli* (ATCC 25922)) bacterial strains, as well as three fungal strains (*A. niger, A. oryzae*, and *Penicillium digitatum),* towards purified ScAI-R and ScAI-L was evaluated by determining the diameter of zone inhibition (mm) around each agar well and the IC_50_ values corresponding to the ScAI-R and ScAI-L concentrations that inhibited the growth of 50% of the bacterial or fungal inoculum, as described in our previous work (Araya and Lomonte, 2007). Commercial ampicillin and cycloheximide were used as reference drugs. The results are the means of three different measurements.

### 3.10 Tumoral cell cytotoxicity

The cell lines HCT 116 and LoVo (human colorectal cancer) and MDA-MB (human breast cancer) were purchased from the American Type Culture Collection (ATCC, United States) and grown in Dulbecco’s Modified Eagles Medium containing 15% fetal bovine serum at 37°C in a 5% CO_2_-humidified incubator for 24 h. The MTT assay was used to assess cell metabolic activity. Cell viability was calculated as the mean ± SD (n = 3) and was expressed as the relative percentage of the OD values measured at 550 nm for α-AI-treated cells. The cell viability determined at 550 nm for ScAI-R and ScAI-L-treated cells and the control was expressed as the relative percentage of the OD values (cell viability ratio (%) = (OD treated/OD untreated) * 100%). The results are presented as the mean ± SD (n = 3). The cells (4 × 10^4^ in each well) were incubated in a 96-well plate at 37°C for 24 h in the absence or presence of different concentrations of ScAI-R and ScAI, previously diluted in the culture medium. Then, 20 µL of MTT (5 mg/mL in PBS) was added to the cells, and the cells were incubated at 37°C for 4 h. Finally, the medium was carefully removed from each well and replaced with an equal volume of saline solution (50:50). To dissolve the formazan crystals, the preparations were mixed thoroughly using a shaker before measuring the absorbance at 550 nm.

## 4 Conclusion

The current study shows that *S. costu*s root and leaf extracts are excellent sources of α-AIs. Based on the biochemical and functional characterization results, the two purified α-AIs (ScAI-R and ScAI-L) could play important roles in the management of, and prophylaxis against, diabetic diseases and obesity disorder. Moreover, these α-AIs are may be protective against bacterial and fungal strains and display cytotoxicity towards human cancer colorectal cells (LoVo and HCT-116), thereby contributing to the host’s defense against serious microbial infections and cancer. Hence, these antimicrobial/antitumoral molecules might also be good therapeutic targeted drugs that act against pathogen microbial infections and colorectal cancer.

## Data Availability

The raw data supporting the conclusions of this article will be made available by the authors, without undue reservation.
