# Corrigendum: Unlocking the therapeutic potential of *Saussurea costus*: purification and functional characterization of α-amylase inhibitors

**DOI:** 10.3389/fbioe.2025.1592365

**Published:** 2025-03-21

**Authors:** Imen Ben Abdelmalek, Tomather A. A. Alhmdi, Abir Ben Bacha, Najeh Krayem

**Affiliations:** ^1^ Department of Biology, College of Science, Qassim University, Buraydah, Saudi Arabia; ^2^ Biochemistry Department, Science College, King Saud University, Riyadh, Saudi Arabia; ^3^ Laboratory of Biochemistry and Enzymatic Engineering of Lipases, National Engineering School of Sfax, University of Sfax, Sfax, Tunisia

**Keywords:** *Saussurea costus*, α-amylase, enzyme inhibition, diabetes mellitus, stability, antibiotic agent, cytotoxicity

In the published article, there was an error in **Affiliation 1**. Instead of “College of Science, Qassim University, Buraydah, Saudi Arabia,” it should be “Department of Biology, College of Science, Qassim University, Buraydah, Saudi Arabia.”

The authors apologize for this error and state that this does not change the scientific conclusions of the article in any way. The original article has been updated.

